# Predictive risk mapping of an environmentally-driven infectious disease using spatial Bayesian networks: A case study of leptospirosis in Fiji

**DOI:** 10.1371/journal.pntd.0006857

**Published:** 2018-10-11

**Authors:** Helen J. Mayfield, Carl S. Smith, John H. Lowry, Conall H. Watson, Michael G. Baker, Mike Kama, Eric J. Nilles, Colleen L. Lau

**Affiliations:** 1 Research School of Population Health, The Australian National University, Canberra, Australia; 2 School of Business, University of Queensland, Brisbane, Australia; 3 School of People, Environment and Planning, Massey University, Palmerston North, New Zealand; 4 Department of Infectious Disease Epidemiology, London School of Hygiene & Tropical Medicine, London, United Kingdom; 5 Department of Public Health, University of Otago, Wellington, New Zealand; 6 Fiji Ministry of Health and Medical Services, Suva, Fiji; 7 Division of Pacific Technical Support, World Health Organization, Suva, Fiji; 8 Program on Infectious Diseases and Humanitarian Emergencies Harvard Humanitarian Institute, Boston, MA, United States of America; Fundacao Oswaldo Cruz, BRAZIL

## Abstract

**Introduction:**

Leptospirosis is a zoonotic disease responsible for over 1 million severe cases and 60,000 deaths annually. The wide range of animal hosts and complex environmental drivers of transmission make targeted interventions challenging, particularly when restricted to regression-based analyses which have limited ability to deal with complexity. In Fiji, important environmental and socio-demographic factors include living in rural areas, poverty, and livestock exposure. This study aims to examine drivers of transmission under different scenarios of environmental and livestock exposures.

**Methods:**

Spatial Bayesian networks (SBN) were used to analyse the influence of livestock and poverty on the risk of leptospirosis infection in urban compared to rural areas. The SBN models used a combination of spatially-explicit field data from previous work and publically available census information. Predictive risk maps were produced for overall risk, and for scenarios related to poverty, livestock, and urban/rural setting.

**Results:**

While high, rather than low, commercial dairy farm density similarly increased the risk of infection in both urban (12% to 18%) and rural areas (70% to 79%), the presence of pigs in a village had different impact in rural (43% to 84%) compared with urban areas (4% to 24%). Areas with high poverty rates were predicted to have 26.6% and 18.0% higher probability of above average seroprevalence in rural and urban areas, respectively. In urban areas, this represents >300% difference between areas of low and high poverty, compared to 43% difference in rural areas.

**Conclusions:**

Our study demonstrates the use of SBN to provide valuable insights into the drivers of leptospirosis transmission under complex scenarios. By estimating the risk of leptospirosis infection under different scenarios, such as urban versus rural areas, these subgroups or areas can be targeted with more precise interventions that focus on the most relevant key drivers of infection.

## Author summary

Leptospirosis is a zoonotic disease responsible for over 60,000 deaths annually and is transmitted from mammal hosts to humans through contact with infected urine. The range of possible hosts and complex environmental factors related to transmission make targeted interventions challenging. We used spatial Bayesian Networks applied to a case study in Fiji to show that livestock exposure and poverty affect the probability of infection differently in rural compared to urban areas. This work illustrates the complexity of leptospirosis transmission drivers in Fiji, and shows how they are affected by the interactions between livestock exposure and other environmental and socio-demographic factors. In doing so, we support previous findings linking the risk of leptospirosis to poverty.

## Introduction

Leptospirosis is a globally occurring zoonotic disease, with an estimated one million severe cases and 60,000 deaths annually [1]. Infection is particularly common in tropical developing countries, with the highest mortality rates found in Oceania (including the Pacific Islands), accounting for an estimated 9.61 deaths per 100,000 people [1–3]. Outbreaks are often associated with extreme weather events such as flooding, and prevalence is expected to increase as these events become more common as a result of climate and global environmental change [4–7]. With lack of resources being a key limitation for health adaptation to climate change in low and medium income countries [8], the ability to efficiently allocate available resources by tailoring interventions is crucial for maximising impact [9].

Infection in humans is caused by contact with infected urine from a mammalian (non-human) host [10]. This can occur via different exposure pathways and risk of infection is affected by numerous interacting environmental, socio-demographic, and behavioural factors. With global environmental and demographic change, these factors could individually, or possibly synergistically, increase the risk of transmission. The relative importance of these factors also varies between places. For example, urbanisation has been identified as a key predictive factor in the risk of transmission in Brazil [11]. Typically, urbanisation in developing countries results in densely populated areas with high poverty levels, poor dwelling construction and low education rates, such as the favelas of Brazil [12]. However, in other countries such as Fiji, urban dwellers have a lower risk of infection than those in rural areas, mostly due to differences in exposure to subsistence livestock animals [13, 14]. Livestock including pigs, cattle and sheep are known to be important reservoirs [2, 10, 15–17], although exposure to these animals differs across communities [14] and even for different individuals within the same communities.

By assessing the estimated risk of leptospirosis infection under different scenarios and for different sub-populations, such as urban versus rural areas, these groups or areas can be targeted with more precise interventions that specifically focus on the key drivers of infection most relevant to them. Common methods used in epidemiology, such logistic regression models, do not easily allow for scenario analysis and in many cases separate models are required for each scenario, retraining the model each time on a subset of the data [15]. Geographically weighted regression models have been used to determine the spatial variation in the relative importance of environmental factors [18], but like standard regression models, they are not designed for scenario analysis.

Bayesian networks (BNs) are a machine learning technique [19] commonly used in creating decision support systems in numerous fields including environmental management [20–22], and health [14, 23, 24]. BNs are better suited than regression models for assessing complex systems and outcomes under different scenarios [14, 25]. Scenario analysis using BNs is facilitated by a graphical interface which allows decision makers to interact directly with the model, define scenarios (including ones with multiple strongly correlated variables) and explore outcomes. Recently, BNs have been integrated with geographic information systems (GIS) to generate decision support systems that include predictive risk maps [20, 22, 26].

In this paper, we use BNs and a linked GIS to produce a predictive risk map of human leptospirosis infection in Fiji. We also examine different scenarios for selected combinations of environmental and livestock exposures to examine how these interactions may impact the risk of disease transmission.

## Materials and methods

### Ethics statement

Ethics approvals were granted by the Fiji National Research Ethics Review Committee (2013 03), the Human Research Ethics Committee of The University of Queensland (2014000008) and the London School of Hygiene & Tropical Medicine (6344). Support was sought and obtained from divisional and sub-divisional Ministry of Health officers for community visits.

### Study location and setting

Fiji is divided into 86 Tikinas (administrative areas), which are further broken down into enumeration areas (EAs) of between 80–120 households. The total population is approximately 837,217 [27] and is predominately iTaukei (native Fijian) (57%), with Fijians of Indian descent (Indo-Fijians) comprising 35% [27]. Livestock are commonly kept for both commercial and subsistence purposes. Contact with specific livestock species varies between ethnic groups and urban/rural settings [14]. Fijians have varying access to education and basic services such as electricity and metered water (treated water supplied to houses), particularly between rural and urban areas.

### Data sources

Data were obtained from an eco-epidemiological study of leptospirosis on the three major islands in Fiji (Viti Levu, Vanua Levu and Taveuni) conducted in 2013, as well as from government departments and the most recent census [13]. In 2013, field data were collected on 2,152 human participants from 82 villages, and included questionnaire data on household-level and village-level risk factors, such as the presence of livestock and other animal species, serological data (using the microscopic agglutination test) indicating evidence of past leptospirosis infection, and GPS coordinates of place of residence. Census and government data included environmental and sociodemographic factors such as rainfall, poverty and education levels as well as information on commercial livestock. This information was available at either the Tikina or EA level. A total of 50 potential predictor variables were identified in this study (Table A in S1 Appendix).

As this study aims to look at environmental rather than individual-risk factors, values for predictor variables and the percentage of the population with antibodies to *Leptospira* (seroprevalence) were summarised to the village level. A full description of the data is given in a previous publication by Lau et al [7].

### Generating spatial data

Data on environmental and socio-demographic predictors were plotted onto maps of Fiji at the Tikina or EA level, depending on the resolution of the dataset. The resulting maps of the predictor variables were clipped to include only areas within 1km of populated places and converted to 50 m grids (raster layers). The grids were then converted to the ASCII format required by the GeoNetica software [28]. Data on the presence of pigs were only available at the village level, so a country-level grid layer was not generated.

### Bayesian networks

BNs combine a graphical interface overlaying a probabilistic data model. In the graphical component, variables are represented as nodes, which are joined by links [19, 25]. The direction of a link implies causality from parent node to child node. Depending on the context, parent nodes can alternatively be considered as indicators (predictor variables).

**Fig 1:**
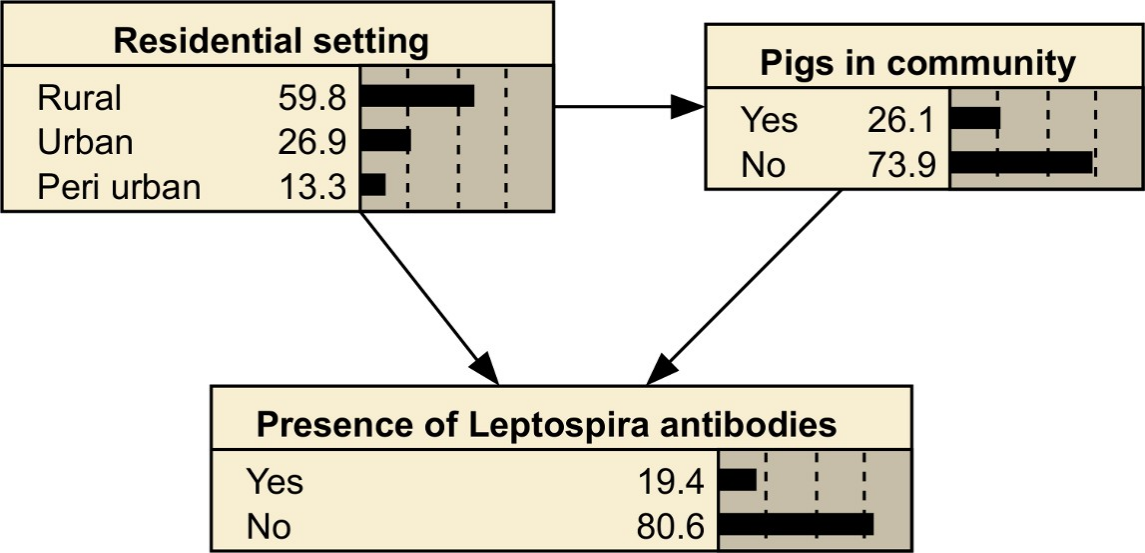
A simple BN showing the probability of the presence of *Leptospira* antibodies being present (target node) is influenced by the residential setting and the presence of pigs in the community. The ‘Residential settings’ node and the ‘Pigs in community’ node are parent nodes of the presence of *Leptospira* antibodies node, which is referred to as the child node. ‘Pigs in community’ is also a child node of ‘Residential setting’.

The dependent variable in an analysis is referred to as the target node in a BN. An example BN is given in Fig 1, with presence of *Leptospira* antibodies as the target node.

The data component of a BN is stored in conditional probability tables (CPTs), in the case of a node linked to parent nodes, or as probability distribution tables for parentless nodes. CPTs contain the probability of a node being in a given state for each combination of the parent nodes. An example CPT for the BN shown in Fig 1 is given in Table **1**.

**Table 1:** Example CPT for the presence of *Leptospira* antibodies node showing the probability of antibodies being present for each combination of residential setting and presence of pigs in the community.

Residential setting	Pigs in community	Presence of Leptospira antibodies
Rural	Yes	27.50%
Rural	No	22.30%
Urban	Yes	23.80%
Urban	No	8.90%
Peri-urban	Yes	25.90%
Peri-urban	No	12.90%

**Fig 2:**
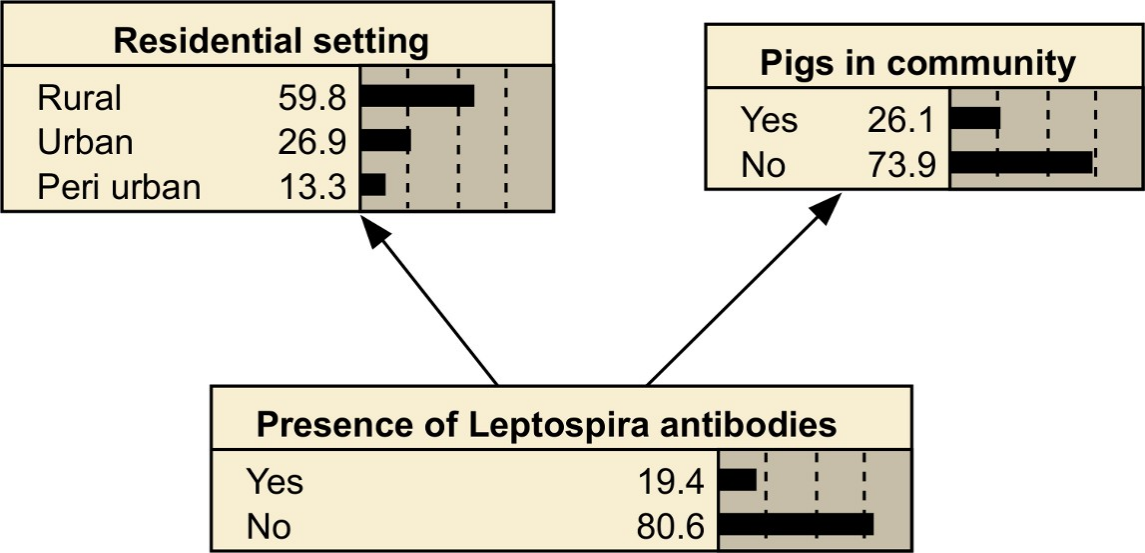
A naïve Bayesian network relating the residential setting and presence of pigs in the community to the probability of Leptospira antibodies.

The simplest form of BN is a naïve BN, in which every node is a child of the target node, and only the target node. A naïve structure has the benefit of relatively small CPTs, but does not account for any interactions between predictor variables (Fig 2).

Although the interpretation of the arrows in Fig 2 is counter-intuitive compared to structures that point from cause to effect (*Leptospira* antibodies do not cause there to be pigs in a community), the child nodes (‘Pigs in community’ and ‘Residential setting’) are being used here as indicators of the parent node. That is, the links in this model represent inference rather than causation.

More structured networks allow more complex links (and interactions) between nodes. Structures can be machine-learned, such as a “tree augmented naïve” (TAN) network, in which every variable has the target node and at most one other node as a parent node [19]; or expert structured, where variables and links are defined by the modeler based on knowledge about disease transmission and/or the research question(s) being asked. Structured networks have been shown to improve the predictive performance of BNs by taking into account the complex interactions between predictor variables, including in a previous study of leptospirosis in Fiji [14]. BNs were implemented in the Netica software [29].

### Categorising predictor variables

Most BN software packages, including Netica [29], require continuous variables to be categorised to form the different states of each node. The suitable number of categories for each variable is influenced by several factors including the amount of data available (i.e. the number of ‘cases’ available for machine learning) and the structure of the network. The size of a CPT is determined by the number of parent nodes, and the number of states in each parent nodes. Models may be unstable if CPTs are too large relative to the size of the dataset [25].

As our study only included 82 cases (villages), each node (variable) was categorised into two states, with approximately 50% of villages in each. To reduce the number of states in the residential setting node, the 12 peri-urban villages, were combined with the urban villages into a single ‘urban’ category. The target node, the Presence of *Leptospira* antibodies, was set to represent the probability that the village’s seroprevalence was below or above the average seroprevalence of 17% found in the study, i.e. a proxy measure of whether each village was below or above average risk.

### Variable selection using naïve network

To generate a parsimonious model, we removed any variables that were not substantially contributing to the predictive performance of the network. To assess this, 50 pairs of data were created from the dataset, for each pair randomly allocating 80% of the data to the training set and the remaining 20% to the testing set. Using a naïve network structure, we carried out a sensitivity to findings analysis in the Netica software package [29]. This analysis lists variables in order of their influence on the target variable. Influence was measured using variance reduction, which describes the expected reduction in the target variable as a result of an observed value of the predictor variable. Predictor variables were removed in order from least to most influential. Each time a variable was removed, CPTs were recalculated using a training dataset (80% of data) and then evaluated based on the predicted values for each case in the corresponding testing set. This evaluation was repeated for each of the 50 training/testing data pairs, each time evaluating the area under the receiver operating curve (AUC) [30] and the true skill statistic (TSS) [31] scores of the network. The procedure was repeated until the TSS and AUC scores of the BN began to deteriorate, after which time no further variables were removed, i.e. exclusion of the remaining variables would have significantly affected model performance.

### Structured network

A TAN structure (learnt using Netica) was used to account for the most important variable interactions. The final model was validated using TSS and AUC over 50 trials using the same training/testing dataset pairs used for variable selection.

### Generating risk maps

GeoNetica [28] is a software program that maps the output of BN. Once a BN has been has been implemented in the Netica software (including CPTs), GeoNetica [28] uses maps of the predictor variables to generate a prediction map for the target variable by setting the states of the nodes for each cell in the map. Where a corresponding predictor map has been included, GeoNetica uses the value of the predictor for that cell to set the state of that node in the BN. The state for a node can also be set directly through the BN, for example, setting the ‘Pigs in village’ node to be ‘Yes’, will set the node state to be ‘Yes’ for every cell in the map. If a node state is not selected, and no map is provided, the value for that node is estimated according to values in either the CPT (for nodes with parents) or the probability distribution tables for parentless nodes.

To minimise spurious predictions caused by lack of data for uncommon scenarios, spatial layers were only included for the most influential nodes based on a sensitivity to findings analysis on the final BN. Where necessary, node states were adjusted to have the same minimum and maximum values as the corresponding GIS dataset. Predictive risk maps were generated for selected areas in each of the three divisions included in the 2013 field study [13], in and surrounding the cities of Suva (Central Division) and Labasa (Northern Division), and the coastal area from Sigatoka to Ba (Western Division). Because of the limited number of data points used to train the model (82 villages), predictive risk mapping was limited to these areas, where we had sufficient data for robust predictions.

### Scenario analysis

**Fig 3:**
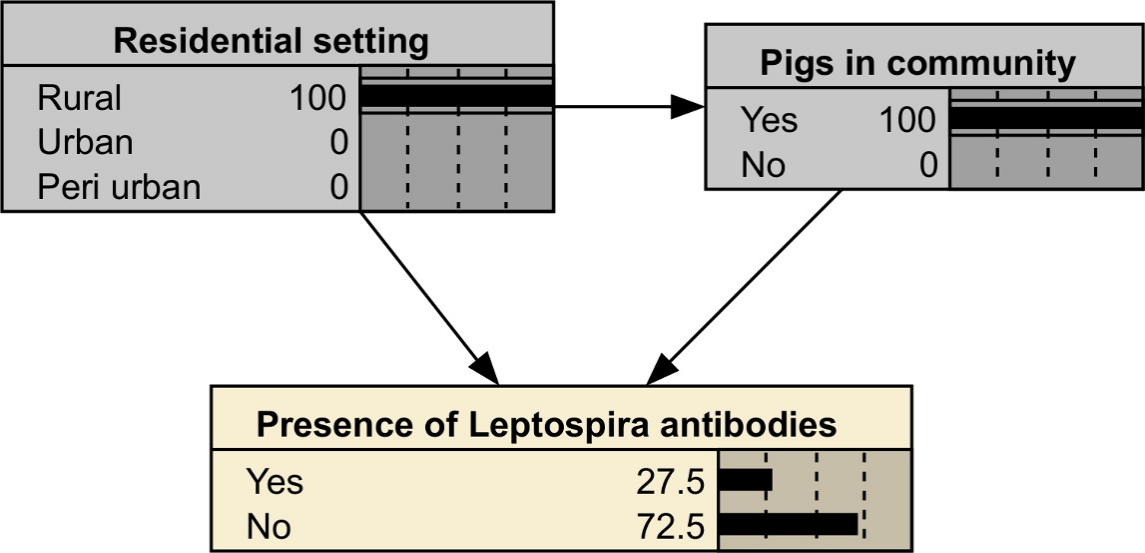
Example BN with the evidence set to show the probability of Leptospira antibodies being present in a rural setting with pigs in villages.

Scenarios were defined by selecting combinations of states for the relevant nodes, and leaving all other nodes in the default state. For example, by setting the ‘Pigs in village node’ to ‘Yes’ and the ‘Residential setting’ node to ‘Rural’, the BN will calculate the probability of the Presence of *Leptospira* antibodies in rural communities with pigs (Fig **3**).

When generating risk maps for different scenarios, the variable being examined was fixed as before, and the variation between urban and rural areas was compared on the map. Individual livestock and poverty scenarios were tested, with each being analysed separately for both urban and rural areas.

## Results

### Variable selection for the final model

The seven most influential predictor variables identified using the naïve network and sensitivity analysis were urbanisation, population density, subsistence farming, primary education or less, tertiary education, households with electricity supply, and percentage of houses with good construction. We removed tertiary education from the final model as it was strongly correlated with primary education (Pearson’s coefficient -0.77), but was the less influential of the two.

In addition to the influential variables identified through sensitivity analysis, poverty rate and the two most influential livestock variables (presence of pigs in the village and commercial dairy farm density) were specifically included in the final model to allow scenario analysis related to these variables. The final nine variables used in the final models are shown in Table 2. See Figure A in S2 Appendix for full results from variable selection.

**Table 2:** Final variables used in predictive model. Variance reduction describes the expected reduction in the probability of above average seroprevalence in village as a result of an observed value of the predictor variable.

Variable	Categories (states in BN)	Variance reduction
Urbanisation [27]	• Rural • Urban & peri-urban	35.30%
Population density [27]	• High (> = 1.3) • Low (< 1.3)	34.30%
Subsistence farming (% of population who depend entirely on subsistence crops) [27]	• High (> = 13%) • Low (<13%)	28.90%
Primary education or less (% of population with primary school education or less) [27]	• High (> = 24%) • Low(<24%)	23.80%
Households with electricity supply (% of households on the Fiji Electric Authority’s supply grid) [27]	• High (> = 89%) • Low(<89%)	21.50%
Percentage of houses with good construction [27]	• High (> = 49%) • Low(<49%)	19.40%
Pigs in village [Questionnaire data, [13]	• Present • Absent	17.60%
Poverty rate (% of population below poverty rate) [32]	• High (> = 39%) • Low(<39%)	11.70%
Dairy farm density (Number of dairy farms/sq km in Tikina) [33]	• High (> = 0.028 farms/sq km) • Low (<0.028 farms/sq km)	3.82%

### Model predictive ability

**Fig 4:**
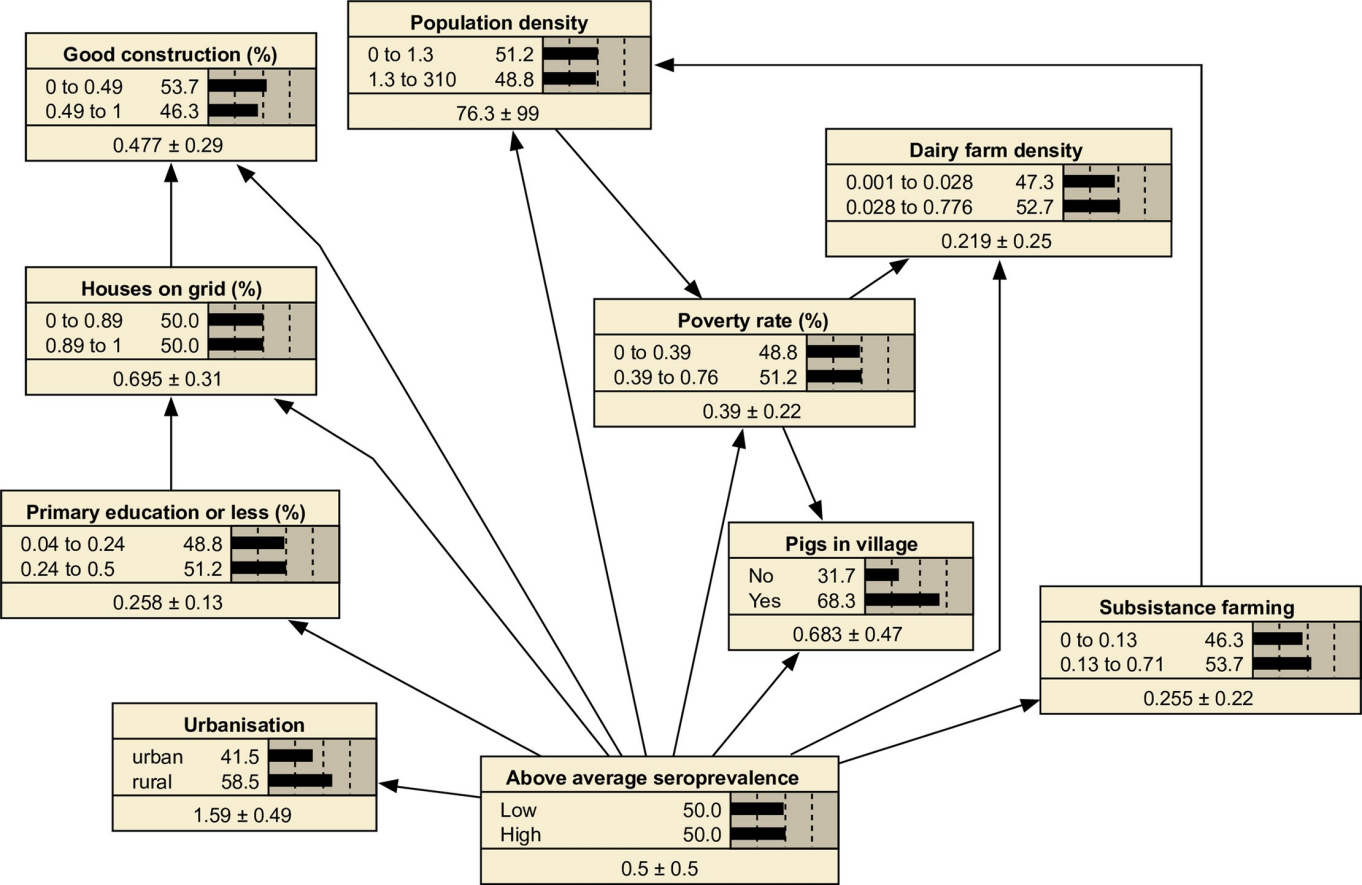
TAN Bayesian network designed to predict the probability of above average seroprevalence in villages in Fiji, with predictor variables shown in their default states (i.e. no scenarios defined). The structure of the network was learnt using the Netica software to account for relationships between predictor variables.

When structured as a TAN, the resulting BN model (Fig 4) had a mean AUC of 0.89 (SD = 0.07) and a mean TSS of 0.64 (SD = 0.19) over 50 trials. This was comparable to a naïve network constructed with the same variables (AUC mean = 0.88, SD = 0.08, TSS mean 0.64, SD 0.18).

### Predictive risk map

**Fig 5:**
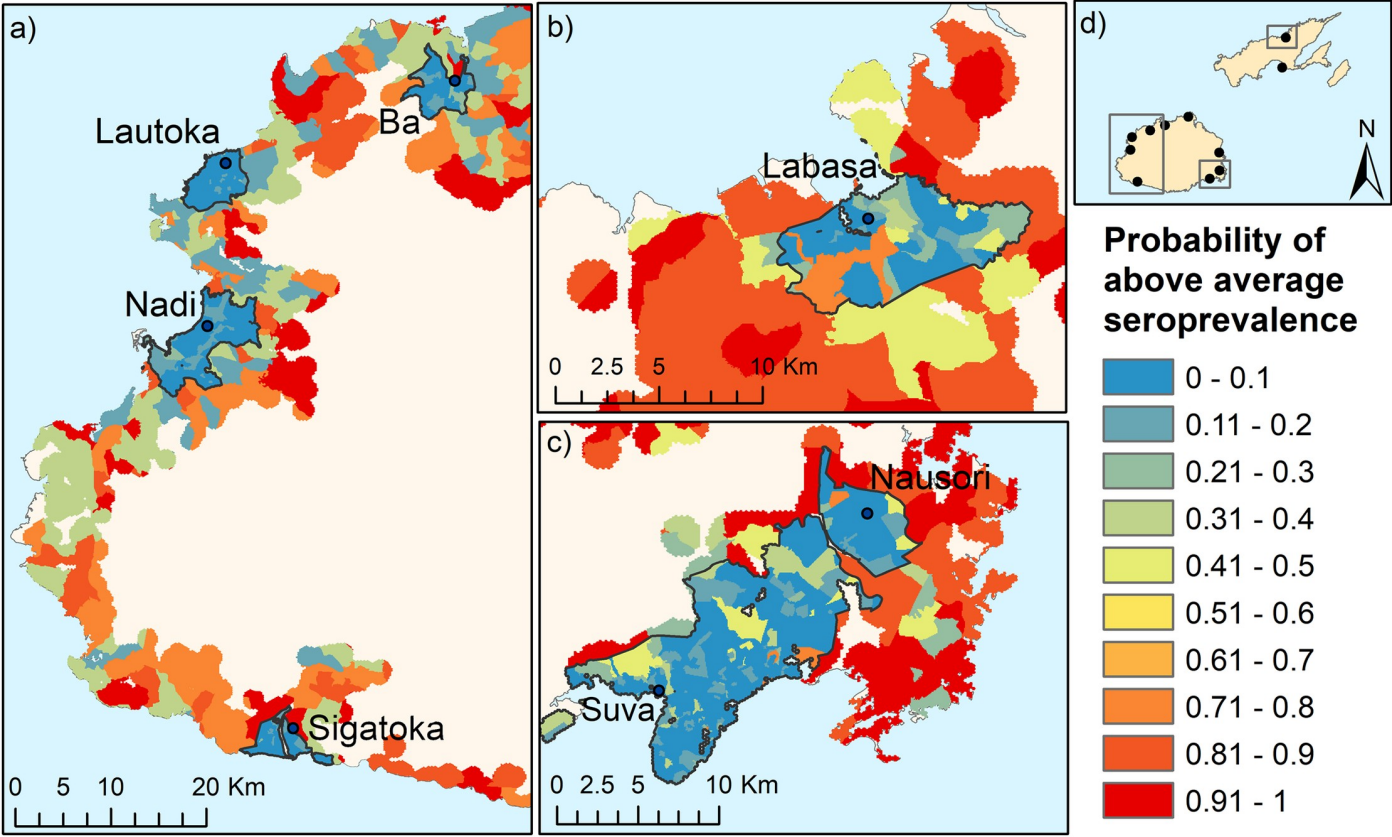
Predictions of probability of above average seroprevalence generated from the BN for selected regions of Fiji: a) Western Division, b) Northern Division, c) Central Division, d) map of Fiji showing approximate locations of the predictive risk maps. Urban and peri urban areas are outlined in black.

Spatial layers were created for the most influential nodes: urbanisation, population density (1000/ha), subsistence farming, percentage of residents with primary education or less, and houses on the electricity grid. Urbanisation and population density had similar spatial distributions (i.e. high population density was found only in urban areas); therefore the spatial layer for population density was not included for predictive risk mapping. Spatial layers for the remaining four nodes were combined with the BN to generate the predicted risk maps. In general, the BNs predicted a much greater chance of above average seroprevalence in rural areas than urban areas, which is clearly evident in the mapped results (Fig 5).

### Scenario analysis

**Fig 6:**
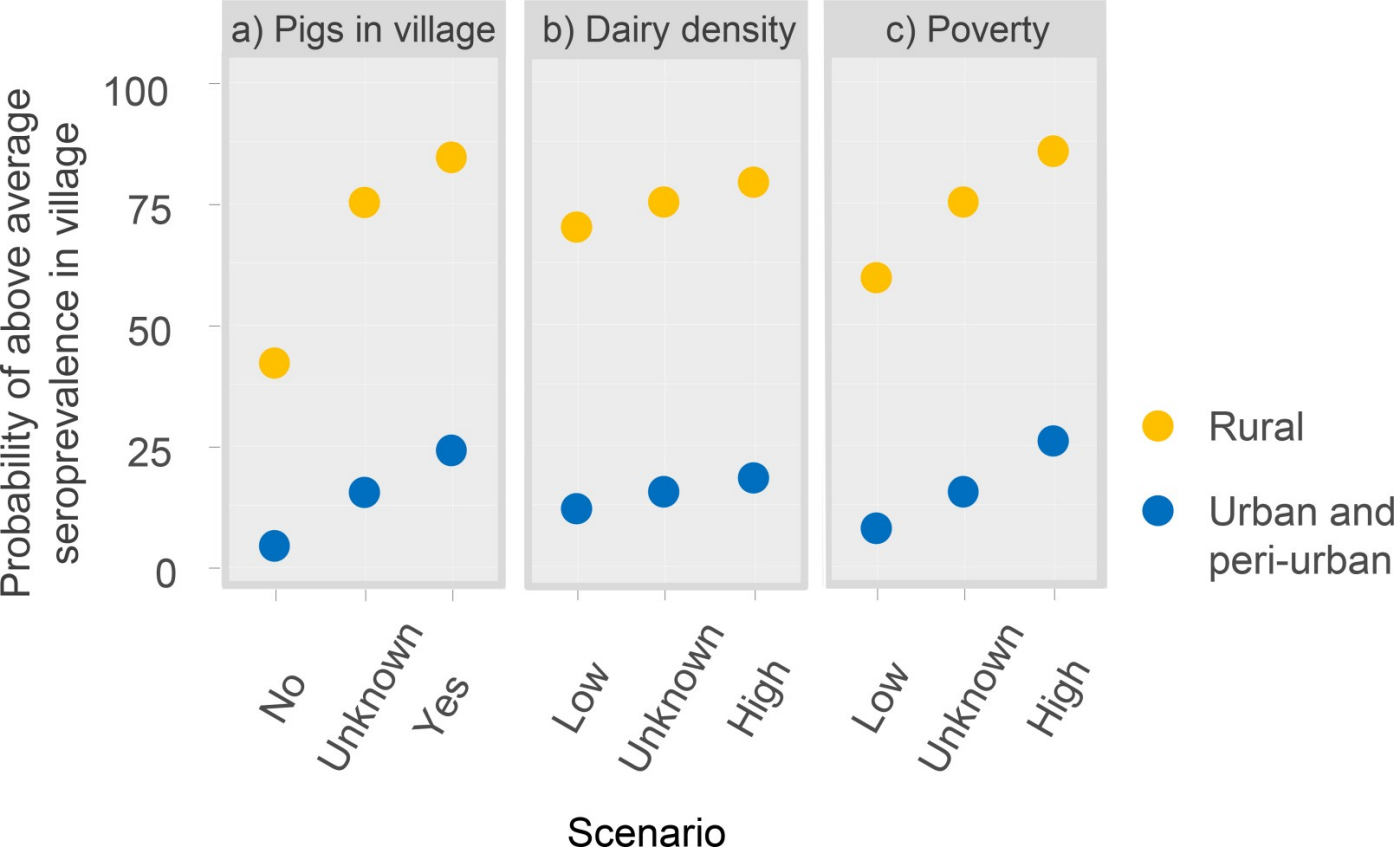
Predictions of probability of above average seroprevalence in urban and rural areas for scenarios based on (a) pigs present in the village, (b) commercial dairy farm density and (c) poverty levels.

Scenario analysis was used to examine whether poverty, the presence of pigs in a village or the density of dairy farms have different impact on the predicted seroprevalence in rural compared to urban areas (Table 3, Fig 6).

**Table 3:** Number of villages in each scenario (total 82 villages from 2013 field study)[13].

	Pigs		Dairy farm		Poverty		Total
	No	Yes	Low	High	Low	High	
Rural	7	41	17	15	17	31	**128**
Urban /peri-urban	19	15	9	13	30	4	**90**
**Total**	**26**	**56**	**26**	**28**	**47**	**35**	

There was a clear difference in the predicted probability of above average seroprevalence in rural compared to urban areas under different scenarios of livestock exposure and poverty rates (Fig 6). The presence (rather than absence) of pigs in a village was more influential in rural rather than urban areas (an increased probability of above average seroprevalance from 41.7% to 84.4% for rural areas compared to an increase from 3.94% to 23.7% for urban areas), however the difference was less marked with low compared to high dairy farm density (an increased probability of above average seroprevalance from 69.7% to 79.2% for rural areas compared to an increase from 11.7% to 18.0% for urban areas). While high and low poverty rates appear to have similar impact on both urban and rural areas, it should be noted that the proportional increase in risk is much higher in the urban settings.

### Risk maps

**Fig 7:**
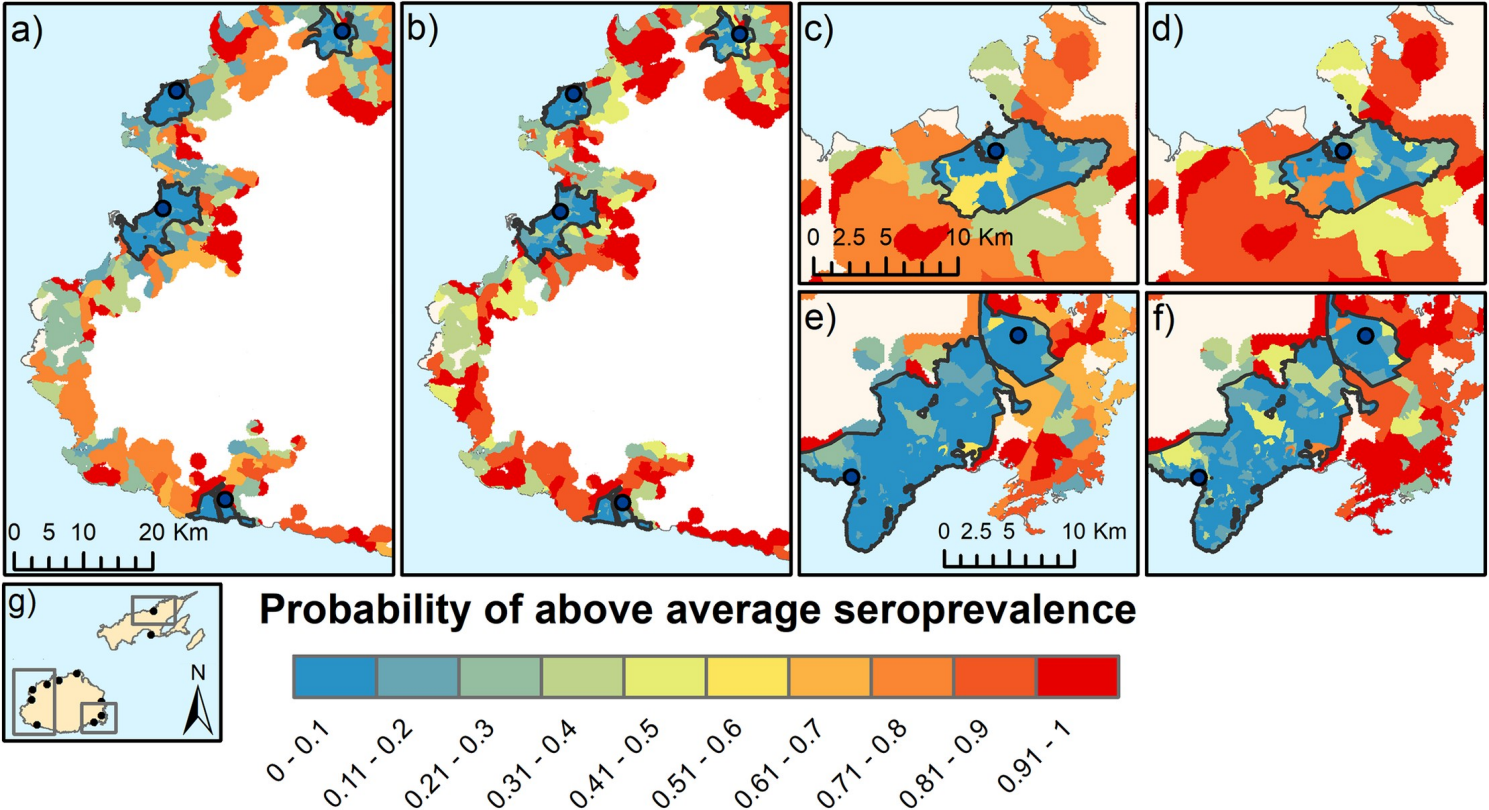
Predictions of probability of above average seroprevalence generated from the spatial BN under different scenarios of commercial dairy farm density a) Western Division, low density, b) Western Division, high density, c) Northern Division, low density d) Northern Division, high density e) Central Division low density, f) Central Division high density, g) map of Fiji showing approximate locations of the predictive risk maps. Urban and peri-urban areas are outlined in black.

Risk maps for different scenarios of commercial dairy farm density show that the increase in predicted risk in low density and high density scenarios are similar in urban and rural areas (Fig 7).

**Fig 8:**
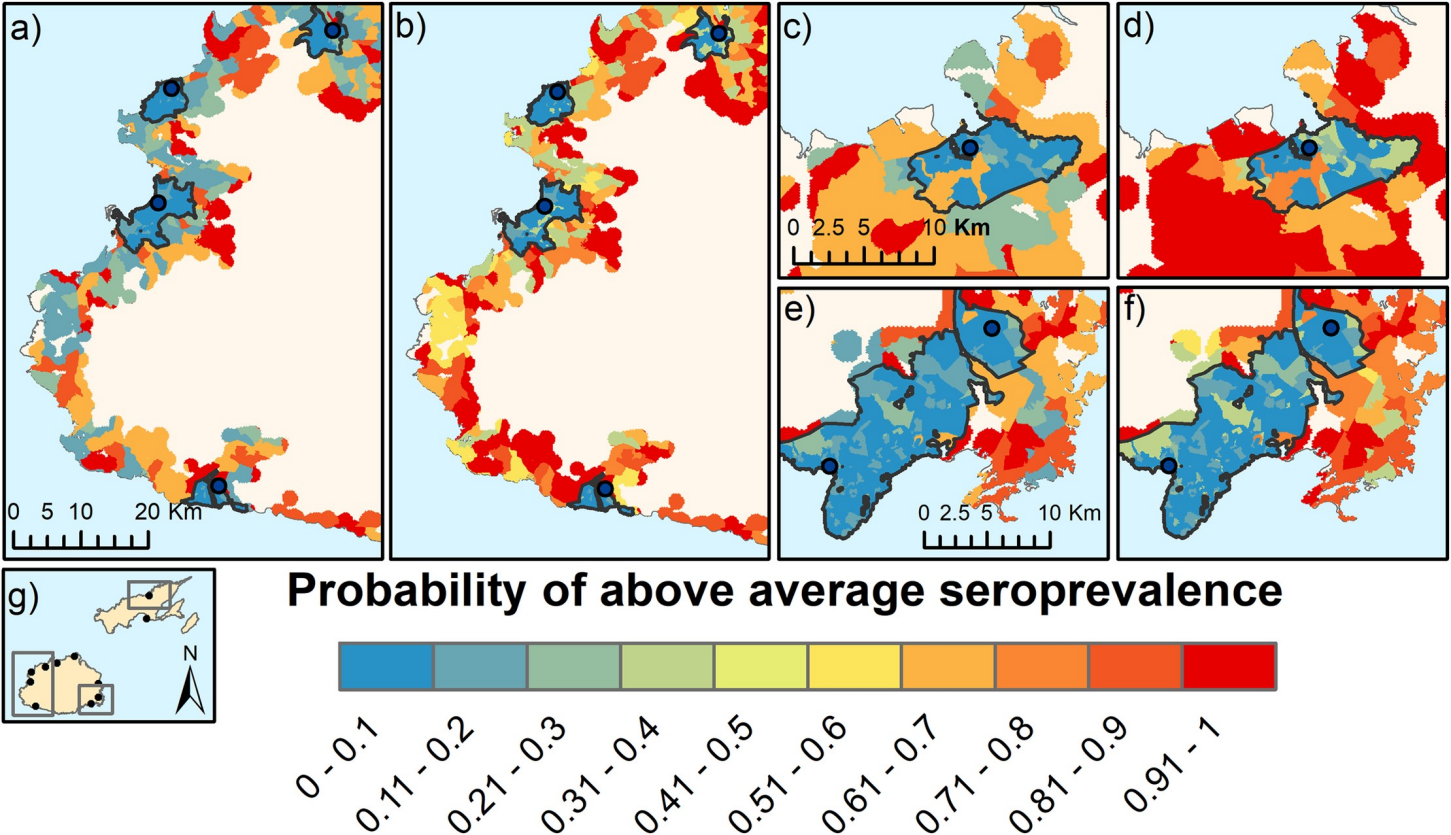
Predictions of probability of above average seroprevalence generated from the BN under different scenarios of poverty rates: a) Western Division, low poverty, b) Western Division, high poverty, c) Northern Division, low poverty d) Northern Division, high poverty e) Central Division low poverty, f) Central Division high poverty, g) map of Fiji showing approximate locations of the predictive risk maps. Urban and peri-urban areas are outlined in black.

Maps of the scenarios for low and high poverty rates showed a predicted increase in probability of above average seroprevalence with high poverty levels in both urban and rural areas (Fig 8).

## Discussion

Our study demonstrates the use of spatial BNs to provide valuable insights into the different drivers of leptospirosis transmission under complex scenarios, and the spatial variation in transmission risk. In Fiji, important environmental and socio-demographic factors included living in rural areas, poverty levels, and livestock exposure. Our results support previous studies that agricultural intensification may contribute to increased outbreaks of zoonotic diseases such as leptospirosis [34]. Although seroprevalence (and therefore infection risk) was higher in rural areas, the impact of livestock exposure was shown to differ between urban and rural areas. However, while high commercial dairy farm density similarly increased the risk of infection in both urban and rural areas, the presence of pigs in a village had a greater impact in rural compared with urban areas.

Urban slums in developing countries have been linked to high risk of many diseases, including leptospirosis [12]. In our study, areas of high poverty rates were predicted to have 26.6% and 18.0% higher probability of above average seroprevalence in rural and urban areas, respectively, compared to areas of low poverty rates. Although the absolute difference in these predictions is relatively small (<8%), they represent >300% difference in urban areas compared to 43% in rural areas. A possible explanation for the greater difference in risk in urban areas is that, particularly in developing countries, the disparity between the rich and the poor is typically much greater in urban areas compared to rural areas, resulting in greater inequities in health. This is particularly evident in urban slums, where marginalized and neglected populations suffer from very poor health outcomes, including infectious diseases [12, 35, 36].

Our results corroborate findings from other studies in diverse settings that leptospirosis is a disease of poverty, especially in urban slums, and disproportionately affects the most vulnerable populations [1, 11]. Population growth and urbanization in developing countries typically result in areas of urban and peri-urban poverty; together with climate change, rising frequency of extreme weather events, and the high risk of flooding in urban slums, both endemic and epidemic leptospirosis are likely to cause an increasing disease burden in the future [4, 35–38].

Of note in this study is that the maximum rainfall did not present as one of the most influential variables, despite the previous links between flooding and leptospirosis outbreaks [4]. There are several possible reasons for this. Firstly, while high rainfall may increase the risk of infection, there may not be sufficient variation in rainfall across the islands of Fiji for this to be an influential predictor variable. Secondly, other environmental factors may influence the effect of rainfall, e.g. in flood prone compared to well drained areas.

By including a spatial component to the BNs, we were able to produce predictive risk maps to demonstrate the spatial variation in the impact of poverty and livestock exposure on leptospirosis risk between urban and rural areas. There are several advantages to using a BN modeling approach compared to the commonly applied regression models for generating risk maps. Firstly, spatial data used for training the models generally do not meet the assumption of independence of a regression models due to spatial autocorrelation [39]. BNs reduce this constraint by allowing relationships between the variables to be accounted for in the model (in this case by using a TAN network structure). Secondly, linking a BN to a GIS allows for multivariable scenarios to be mapped out without needing to retrain the model on a subset of the data.

In this work, when the scenarios for high or low dairy farm density were mapped, differences in the amount of increased risk between urban and rural areas became apparent. The substantially lower risk posed by living in an urban compared to rural setting is also evident in the mapped visualization of the predictions. This study demonstrates the utility of spatial BN for analyzing outcomes under different scenarios. The integration of BNs with spatial data allows spatially explicit scenarios to be examined more easily than with traditional regression methods.

Despite the benefits of a BN approach, one caveat that should be considered is the possible loss of information as a result of discretising the variables to form the states of the nodes. In this study, nodes were discretised into only two states in order to minimise the number of combinations that occur when generating the risk map (i.e. every when node is set to a particular state). This helps to reduce the uncertainty that arises from incomplete CPT tables. By doing this, it is possible that relationships between the variables may not have been detected. In situations with either more data or fewer scenarios, a larger number of states may be appropriate.

In this study, we focused on a few selected scenarios as a case study to demonstrate the utility of spatial BNs for understanding leptospirosis transmission, but a wide range of other scenarios, including more complex ones, could be explored. Because this study was based on a dataset of only 82 villages, we limited each variable to two states to ensure robustness of the model. Larger datasets would allow more refined classifications of predictor variables, and potentially provide further insights into more complex scenarios, including scenarios that include combinations of states of urban/peri-urban/rural *and* poverty rates *and* animal exposure.

Our study provides empirical evidence that the drivers of leptospirosis transmission in Fiji are complex, and include environmental and socio-demographic factors, as well as exposure to livestock. This information supports a One Health approach to disease prevention and control that takes into account human, animal, and environmental factors. Our findings also suggest that to achieve maximum impact, a more targeted and precise approach to public-environmental health strategies is needed, where interventions are specifically designed for specific scenarios. Spatial BNs can be used to help pinpoint hotspots and identify the most important drivers of transmission in different areas. Future studies should also be specifically designed to assess the impact of interventions under different scenarios.

## Supporting information

S1 Appendix Full list of variables considered. List of potential predictor variables in order of their influence on the probability of above average leptospirosis, with variable 1 (Urbanisation) having the most influence.
(DOCX)

S2 Appendix Selection of variables for Bayesian networks. Average AUC and TSS scores over 50 trials using a naïve Bayesian network. Variables are sequentially removed, starting from model 50.
(DOCX)
