# Supplementary material for: Predictive risk mapping of an environmentally-driven infectious disease using spatial Bayesian networks: A case study of leptospirosis in Fiji
Source: PLoS Negl Trop Dis. 2018 Oct 11;12(10):e0006857. doi: 10.1371/journal.pntd.0006857 (PMC6198991; doi:10.1371/journal.pntd.0006857)
Supplement: S1 Appendix — List of potential predictor variables in order of their influence on the probability of above average leptospirosis, with variable 1 (Urbanisation) having the most influence. (DOCX) [file pntd.0006857.s001.docx]

**S1: Full list of variables considered**

**Table A:** List of potential predictor variables in order of their influence on the probability of above average leptospirosis, with variable 1 (Urbanisation) having the most influence

| **Influence** | **Variable** | **Scale** | **source** |
| --- | --- | --- | --- |
| 1 | Urbanisation | Enumeration area | Fiji National Census 2007 [1] |
| 2 | Population density | Enumeration area | Fiji National Census 2007 [1] |
| 3 | Subsistence farming | Enumeration area | Fiji National Census 2007 [1] |
| 4 | Primary education or less | Enumeration area | Fiji National Census 2007 [1] |
| 5 | Tertiary education | Enumeration area | Fiji National Census 2007 [1] |
| 6 | Houses on electricity grid | Enumeration area | Fiji National Census 2007 [1] |
| 7 | Houses with good construction | Enumeration area | Fiji National Census 2007 [1] |
| 8 | Pigs in the village | Village | Questionnaire [2] |
| 9 | Access to flush toilets | Enumeration area | Fiji National Census 2007 [1] |
| 10 | Distance to river | 1:50K topographic maps | Fiji Ministry of Lands and Resources. Digital data from Fiji Land Information system [3] |
| 11 | Cows in the village | Village | Questionnaire [2] |
| 12 | Poultry density | Village | Questionnaire [2] |
| 13 | Houses with metered water | Enumeration area | Fiji National Census 2007 [1] |
| 14 | Poverty rate | Tikina | Word bank 2011 report[4] |
| 15 | Region | Region | Standard administration boundary |
| 16 | Horses in the village | Village | Questionnaire [2] |
| 17 | Percent iTaukei | Enumeration area | Fiji National Census 2007 [1] |
| 18 | Distance to main road | 1:50K topographic maps | Fiji Ministry of Lands and Resources. Digital data from Fiji Land Information system[3] |
| 19 | Goats in the village | Village | Questionnaire [2] |
| 20 | Cane fields in village | Village | Questionnaire [2] |
| 21 | Commercial dairy farm density | Tikina | Fiji National Agricultural Census 2009[5] |
| 22 | Sheep farm density | Village | Questionnaire [2] |
| 23 | Pig farm density | Village | Questionnaire [2] |
| 24 | Horse density | Tikina | Fiji National Agricultural Census 2009[5] |
| 25 | Sheep in the village | Village | Questionnaire [2] |
| 26 | Population change from 86-07 | Enumeration area | Fiji National Census 2007 [1] |
| 27 | Commercial beef density | Tikina | Fiji National Agricultural Census 2009[5] |
| 28 | Subsistence beef density | Tikina | Fiji National Agricultural Census 2009[5] |
| 29 | Poultry farm density | Tikina | Fiji National Agricultural Census 2009[5] |
| 30 | Cattle density | Tikina | Fiji National Agricultural Census 2009[5] |
| 31 | Minimum rainfall | 100 m | Landcare Research Institute, NZ.[6] |
| 32 | Cattle farm density | Tikina | Fiji National Agricultural Census 2009[5] |
| 33 | Poultry and Duck density | Tikina | Fiji National Agricultural Census 2009[5] |
| 34 | Vegetables grown in village | Village | Questionnaire [2] |
| 35 | Subsistence beef density | Tikina | Fiji National Agricultural Census 2009[5] |
| 36 | Horse density | Tikina | Fiji National Agricultural Census 2009[5] |
| 37 | Commercial dairy cow density | Tikina | Fiji National Agricultural Census 2009[5] |
| 38 | Pig density | Tikina | Fiji National Agricultural Census 2009[5] |
| 39 | Maximum rainfall | 100 m | Landcare Research Institute, New Zealand[6] |
| 40 | Commercial beef farm density | Tikina | Fiji National Agricultural Census 2009[5] |
| 41 | Sheep density | Tikina | Fiji National Agricultural Census 2009[5] |
| 42 | Duck density | Tikina | Fiji National Agricultural Census 2009[5] |
| 43 | Goat farm density | Tikina | Fiji National Agricultural Census 2009[5] |
| 44 | Subsistence dairy farm density | Tikina | Fiji National Agricultural Census 2009[5] |
| 45 | Elevation | 25 m | Landcare Research institute[6] |
| 46 | Slope | 25 m | Landcare Research institute[6] |
| 47 | Goat density | Tikina | Fiji National Agricultural Census 2009[5] |
| 48 | Distance to poorly drained soils | 25 m | Fiji Ministry of Agriculture. 1980/85 National Soil Survey [7] |
| 49 | Secondary education | Enumeration area | Fiji National Census 2007 [1] |
| 50 | Subsistence dairy cow density | Tikina | Fiji National Agricultural Census 2009[5] |

**References**

1. Fiji Bureau of Statistics. *Census of Population and Housing*. 2007 29/06/2017]; Available from: <http://www.statsfiji.gov.fj/index.php/2007-census-of-population>.

2. Lau, C.L., et al., *Human Leptospirosis Infection in Fiji: An Eco-epidemiological Approach to Identifying Risk Factors and Environmental Drivers for Transmission.* PLOS Neglected Tropical Diseases, 2016. **10**(1): p. e0004405.

3. Republic of Fiji Islands. Ministry of Lands and Mineral Resources DoL., *Digital Data from Fiji Land Information System (Flis). GIS Data of 1:50k Topographic Maps*.

4. World Bank, *Republic of Fiji Poverty Trends, Profiles and Small Area Estimation (Poverty Maps) in Republic of Fiji (2003–2009)*. Washington, DC.

5. Fiji Ministry of Agriculture, *Fiji National Agricultural Census 2009*. 2009.

6. Barker G, P.R., *Environmental and Biogeographic Classifications as Spatial Frameworks for Assessing Representativeness in Island Archipelagos: A Fijian Case Study. Hamilton, New Zealand*. Landcare Research.

7. Republic of Fiji Islands. Ministry of Agriculture, *National Soil Survey, GIS Data of Soils and Land Use/Cover.*
