# Supplementary material for: Predictive risk mapping of an environmentally-driven infectious disease using spatial Bayesian networks: A case study of leptospirosis in Fiji
Source: PLoS Negl Trop Dis. 2018 Oct 11;12(10):e0006857. doi: 10.1371/journal.pntd.0006857 (PMC6198991; doi:10.1371/journal.pntd.0006857)
Supplement: S2 Appendix — Average AUC and TSS scores over 50 trials using a naïve Bayesian network. Variables are sequentially removed, starting from model 50. (DOCX) [file pntd.0006857.s002.docx]

**S2: Selection of variables for Bayesian networks**

**Mean score over 50 trials**

**Network ID**

**Fig A:** Average AUC and TSS scores over 50 trials using a naïve Bayesian network. Variables are sequentially removed, starting from model 50. The Network ID denotes which variable from Table one was removed in the following trial. For example, Network 50 contains all 50 predictor variables. Network 48 contains all variables except *Subsistence dairy cow density* (removed in model 49) and *Secondary education* (removed in model 48). See Table A in S1 Appendix for full ordering of variables
